# Supplementary material for: MicroRNA-30b Is Both Necessary and Sufficient for Interleukin-21 Receptor-Mediated Angiogenesis in Experimental Peripheral Arterial Disease
Source: Int J Mol Sci. 2021 Dec 27;23(1):271. doi: 10.3390/ijms23010271 (PMC8745227; doi:10.3390/ijms23010271)
Supplement: Supplementary file 1 [file ijms-23-00271-s001.zip › ijms-1481206-Supplementary.pdf]

Supplemental Table S1. Gene significantly regulated by IL-21R Fc chimera in ischemic mouse muscle

gene	Fold	p_value	q_value
Fosb	0.176	0.000	0.008
1810032O08Rik,Snord1a,Snord1b	0.382	0.000	0.008
Ctgf	0.388	0.000	0.008
Dusp8	0.415	0.000	0.008
Gzma	0.481	0.000	0.008
Ly6c2	0.486	0.000	0.025
Slc10a6	0.492	0.001	0.081
Agtr2	0.494	0.000	0.008
Cxcl9	0.494	0.000	0.008
Pydc3	0.497	0.000	0.008
Otud1	0.501	0.000	0.008
Iigp1	0.501	0.000	0.008
Ces1d	0.504	0.000	0.008
Gbp11,Gbp6	0.506	0.000	0.008
Mx1	0.507	0.000	0.008
Serpina3f,Serpina3g	0.510	0.000	0.008
Il2rb	0.511	0.000	0.008
Gm12250	0.512	0.000	0.008
Chrne	0.512	0.000	0.008
Olfr558	0.515	0.000	0.008
Tlr12	0.520	0.000	0.039
Pydc4	0.530	0.000	0.008
Apoc1	0.533	0.000	0.014
Gimap3	0.538	0.000	0.030
I830012O16Rik,Ifit3	0.541	0.000	0.008
Frzb	0.544	0.000	0.014
Ncr1	0.545	0.001	0.068
Gbp5	0.546	0.000	0.008
Slfn1	0.547	0.000	0.008
Scd1	0.547	0.000	0.008
Gm4951	0.550	0.000	0.008
Zbtb16	0.558	0.000	0.008
Rsad2	0.559	0.000	0.008
AW112010	0.567	0.000	0.008
Isg15	0.571	0.000	0.014
Cmpk2	0.581	0.000	0.008
Ifi47,Olfr56	0.584	0.001	0.081
Irf7	0.587	0.000	0.008
Slamf8	0.588	0.000	0.019

Mrap	0.589	0.001	0.087
Egr2	0.591	0.000	0.008
Cntfr	0.591	0.001	0.093
Ifit2	0.591	0.000	0.008
Oasl1	0.599	0.000	0.008
Glb1l2	0.604	0.000	0.008
Plin1	0.604	0.000	0.008
Cdo1	0.606	0.000	0.008
Btg2	0.606	0.000	0.008
Ifit1	0.610	0.000	0.008
Gvin1	0.612	0.000	0.014
Usp18	0.612	0.000	0.008
Ide	0.613	0.000	0.008
Btaf1	0.613	0.000	0.008
Klrk1	0.614	0.000	0.043
Fasn	0.615	0.000	0.008
Pcx	0.616	0.000	0.008
Ifi27l2a	0.616	0.000	0.008
Cyr61	0.616	0.000	0.008
Gbp2	0.618	0.000	0.008
Card11	0.618	0.001	0.071
Pck1	0.621	0.000	0.008
Thrsp	0.626	0.000	0.008
Nlrc5	0.634	0.000	0.008
Mx2	0.643	0.000	0.019
Oas2	0.645	0.000	0.008
Acss3	0.647	0.001	0.087
Ephx2	0.648	0.000	0.008
Itga8	0.649	0.001	0.056
Oasl2	0.652	0.000	0.008
Ciita	0.652	0.000	0.025
H2-Q7	0.659	0.001	0.068
Zfp612	0.660	0.001	0.056
Gbp3	0.661	0.000	0.008
Rtp4	0.664	0.000	0.008
Zbp1	0.666	0.000	0.047
Siglec1	0.666	0.000	0.008
Lgals12	0.667	0.001	0.052
Cd1d1	0.668	0.000	0.025
Dusp16	0.675	0.000	0.008
Pyhin1	0.682	0.000	0.008
Col6a6	0.682	0.000	0.025
Cd83	0.683	0.000	0.008

Per1	0.683	0.000	0.008
Slc36a2	0.688	0.001	0.062
Adcy5	0.691	0.000	0.039
Pclo	0.692	0.001	0.056
Arhgap15	0.694	0.000	0.043
Ms4a4c	0.694	0.001	0.087
Gm6904,Phf11a,Phf11b,Phf11d	0.696	0.000	0.019
Oas3	0.697	0.000	0.008
Gbp7	0.700	0.000	0.008
Lrrc30	0.701	0.000	0.008
Dusp1	0.701	0.000	0.008
Nfil3	0.702	0.000	0.030
Irgm1	0.704	0.000	0.014
Igtp,Irgm2	0.707	0.000	0.008
Stat2	0.708	0.000	0.014
Slc25a25	0.708	0.001	0.065
Ky	0.709	0.000	0.014
Ifi44	0.710	0.001	0.095
Myh11	0.711	0.000	0.008
Filip1l	0.715	0.000	0.030
Fam13a	0.715	0.001	0.068
Abca8a	0.716	0.000	0.008
Itih5	0.720	0.000	0.008
Tap1	0.722	0.000	0.019
H2-Aa	0.723	0.000	0.008
Trim34a	0.724	0.001	0.098
Spry4	0.730	0.001	0.084
Herc6	0.731	0.000	0.047
Adipoq	0.733	0.000	0.030
Tceal7	0.736	0.000	0.014
H2-Ab1	0.740	0.000	0.019
H2-Eb1	0.741	0.000	0.025
Cidec	0.743	0.001	0.062
Errfi1	0.744	0.000	0.019
Mir1932	0.745	0.001	0.074
Slfn2	0.755	0.001	0.059
Helz2	0.755	0.000	0.014
Tfrc	0.757	0.001	0.062
Ifih1	0.757	0.000	0.039
Nrk	0.758	0.001	0.098
Trim30a	0.760	0.001	0.068
Plagl1	0.760	0.001	0.062
Fcgr1	0.760	0.001	0.059

Bst2	0.761	0.001	0.052
Clec7a	0.761	0.001	0.078
Ddx60	0.761	0.001	0.081
Pld4	0.766	0.001	0.068
Fzd4	0.768	0.001	0.052
Peg10	0.781	0.001	0.095
Ltbp2	1.288	0.001	0.093
Abi3bp	1.291	0.001	0.095
Cthrc1	1.299	0.001	0.091
Pdgfra	1.302	0.000	0.034
Kcnj2	1.310	0.001	0.065
Igfbp3	1.321	0.000	0.043
Mt2	1.325	0.001	0.062
Sfrp1	1.328	0.000	0.043
Medag	1.331	0.001	0.059
Lox	1.334	0.000	0.025
Gda	1.338	0.000	0.019
Ccl9	1.341	0.000	0.047
Col27a1	1.341	0.000	0.047
Kif26b	1.348	0.001	0.056
Col12a1	1.351	0.001	0.078
Aebp1	1.363	0.000	0.030
Thbs4	1.371	0.000	0.008
Ptpn3	1.383	0.001	0.059
Sod3	1.390	0.000	0.019
Itgb3	1.407	0.000	0.034
Col11a1	1.410	0.000	0.008
Itga11	1.416	0.000	0.008
Lrrc15	1.419	0.000	0.008
Tmem8	1.428	0.000	0.019
Sned1	1.428	0.001	0.071
Pcsk5	1.429	0.000	0.034
Mmp19	1.436	0.001	0.071
Ppp1r27	1.437	0.001	0.062
Nfatc4	1.442	0.000	0.008
Cpxm2	1.449	0.000	0.008
Srxn1	1.451	0.000	0.008
Nxpe5	1.463	0.000	0.034
Comp	1.479	0.001	0.093
Sfrp2	1.507	0.000	0.008
Itgbl1	1.513	0.000	0.008
Bahcc1	1.524	0.000	0.008
Ednrb	1.542	0.000	0.008

Cd163	1.550	0.001	0.087
Pi16	1.550	0.000	0.030
Aldh1a3	1.562	0.001	0.065
Glis3	1.563	0.000	0.008
Serpina3k,Serpina3m,Serpina3n	1.566	0.000	0.008
Slc40a1	1.572	0.000	0.008
Stc1	1.579	0.001	0.074
Mmp9	1.583	0.000	0.008
Ptgfr	1.584	0.001	0.074
Rn45s	1.612	0.000	0.008
Myh7	1.624	0.001	0.062
Crlf1	1.637	0.000	0.014
Megf6	1.659	0.000	0.008
Ier5l	1.690	0.000	0.047
Il1rl1	1.730	0.000	0.014
Clec4d	1.866	0.000	0.008
Scn4b	1.915	0.000	0.008
Cyp26b1	1.932	0.000	0.008
Erdr1	1.938	0.000	0.008
Chl1	1.982	0.000	0.008
Ankrd2	2.120	0.000	0.008
Ibsp	2.168	0.000	0.039
Hba-a1,Hba-a2	2.188	0.000	0.008
Wisp2	2.333	0.000	0.008
Beta-s	2.560	0.000	0.008
Hbb-b1,Hbb-b2	2.648	0.000	0.008
Hmox1	2.771	0.000	0.008
Hba-a1,Hba-a2	2.986	0.000	0.008
Acan	3.033	0.000	0.008
Arg1	3.099	0.000	0.008
Tnn	3.490	0.000	0.008
Myl3	3.550	0.000	0.008
